# Multidrug Efflux Systems in *Helicobacter cinaedi*

**DOI:** 10.3390/antibiotics1010029

**Published:** 2012-11-21

**Authors:** Yuji Morita, Junko Tomida, Yoshiaki Kawamura

**Affiliations:** Department of Microbiology, School of Pharmacy, Aichi Gakuin University / 1-100 Kusumoto, Chikusa, Nagoya, Aichi 464-8650, Japan; Email: yujmor@dpc.agu.ac.jp (Y.M.); jtomida@dpc.agu.ac.jp (J.T.)

**Keywords:** *Helicobacter cinaedi*, efflux, antimicrobial resistance

## Abstract

*Helicobacter cinaedi* causes infections, such as bacteremia, diarrhea and cellulitis in mainly immunocompromised patients. This pathogen is often problematic to analyze, and insufficient information is available, because it grows slowly and poorly in subculture under a microaerobic atmosphere. The first-choice therapy to eradicate *H. cinaedi* is antimicrobial chemotherapy; however, its use is linked to the development of resistance. Although we need to understand the antimicrobial resistance mechanisms of *H. cinaedi*, unfortunately, sufficient genetic tools for *H. cinaedi* have not yet been developed. In July 2012, the complete sequence of *H. cinaedi* strain PAGU 611, isolated from a case of human bacteremia, was announced. This strain possesses multidrug efflux systems, intrinsic antimicrobial resistance mechanisms and typical mutations in *gyrA* and the 23S rRNA gene, which are involved in acquired resistance to fluoroquinolones and macrolides, respectively. Here, we compare the organization and properties of the efflux systems of *H. cinaedi* with the multidrug efflux systems identified in other bacteria.

## 1. Introduction

*Helicobacter cinaedi* is a motile, Gram-negative, spiral bacterium belonging to the enterohepatic group of *Helicobacter* species of genus *Helicobacter* (the other group consists of gastric *Helicobacter* species, whose most well-known representative is the infamous *H. pylori*) [1]. During the last two decades, this bacterium has increasingly been recognized as a human pathogen that causes infections such as bacteremia, diarrhea and cellulitis in mainly immune-compromised patients and occasionally in immunocompetent ones with a high potential for recurrence [2,3]. A possible association between *H. cinaedi* and atrial arrhythmias and atherosclerosis was also suggested [4]. 

This pathogen grows slowly over several days on blood agar, even at its optimal conditions, such as a wet microaerobic atmosphere at 37 °C, and often appears as a swarming thin film that is difficult to observe [1,5]. Therefore, it is often problematic to isolate, detect and sub-culture [5,6]. Antimicrobial chemotherapy has been used successfully to treat such infections, but prolonged courses of multiple antimicrobials for at least 2–3 weeks may be required [1]. Recently, molecular epidemiological analysis in Japan showed that all *H. cinaedi* isolates since 2000 had acquired resistance to clarithromycin (macrolides) and ciprofloxacin (quinolones), for which the MIC_90_ (μg/mL) was >128 and 128, respectively, and contained typical mutations in *gyrA* and the 23S rRNA gene, respectively [7,8]. Unlike *H. pylori*, enteric *Helicobacter* species, such as *H. cinaedi*, are intrinsically resistant to amoxicillin (penicillin) [9]. High-level resistance and intrinsic resistance often require the presence of endogenous multidrug efflux pumps [10,11], which have not yet been analyzed in *H. cinaedi*. 

## 2. Multidrug Efflux Systems in Bacteria

Multidrug efflux transporters are fundamental antimicrobial resistance mechanisms in Gram-negative bacteria [12]. Multidrug efflux transport has been studied extensively in bacteria, including ε-proteobacteria, such as *H. pylori* and *Campylobacter jejuni*, but not *H. cinaedi* [e.g., 13,14]. Most bacterial multidrug efflux pumps function as secondary transporters coupled with the proton-motive force (e.g., AcrB of *Escherichia coli* [15], MdfA of *E. coli* [16], and EmrE of *E. coli* [17]) and, although very rare, the sodium-motive force (e.g., NorM of *Vibrio parahaemolyticus* [18]), while some pumps hydrolyze ATP (e.g., MacB of *E. coli* [19] and VcaM of *Vibrio cholerae* [20]). Multidrug efflux transporters can be single component transporters that act at the cytoplasmic membrane (e.g., MdfA and EmrE of *E. coli* and NorM of *V. parahaemolyticus*) in both Gram-negative and -positive bacteria or three component transporters that span the entirety of the Gram-negative cell envelope (e.g., AcrAB-TolC of *E. coli* and MexXY-OprM of *Pseudomonas aeruginosa* [21]), *i.e.*, cytoplasmic membrane transporter component (e.g., AcrB and MexY), outer membrane factor (OMF) component (e.g., TolC and OprM) and periplasmic component belonging to the membrane fusion protein (MFP) family (e.g., AcrA and MexX) [13,14]. Although bacterial multidrug efflux transporters fall into five families, *i.e.*, resistance nodulation cell division (RND) (e.g., AcrB of *E. coli* and MexY of *P. aeruginosa*), major facilitator (MF) (e.g., MdfA of *E. coli*), small multidrug resistance (SMR) (e.g., EmrE of *E. coli*), multi-antimicrobial and toxic extrusion (MATE) (e.g., NorM of V*. parahaemolyticus*) and ABC (ATP binding cassette) (e.g., VcaM of *V. cholerae*)), the RND family is the most clinically relevant in Gram-negative bacteria [14].

## 3. Antibacterial Resistance Revealed by the Complete Genome of *H. cinaedi*

**Figure 1:**
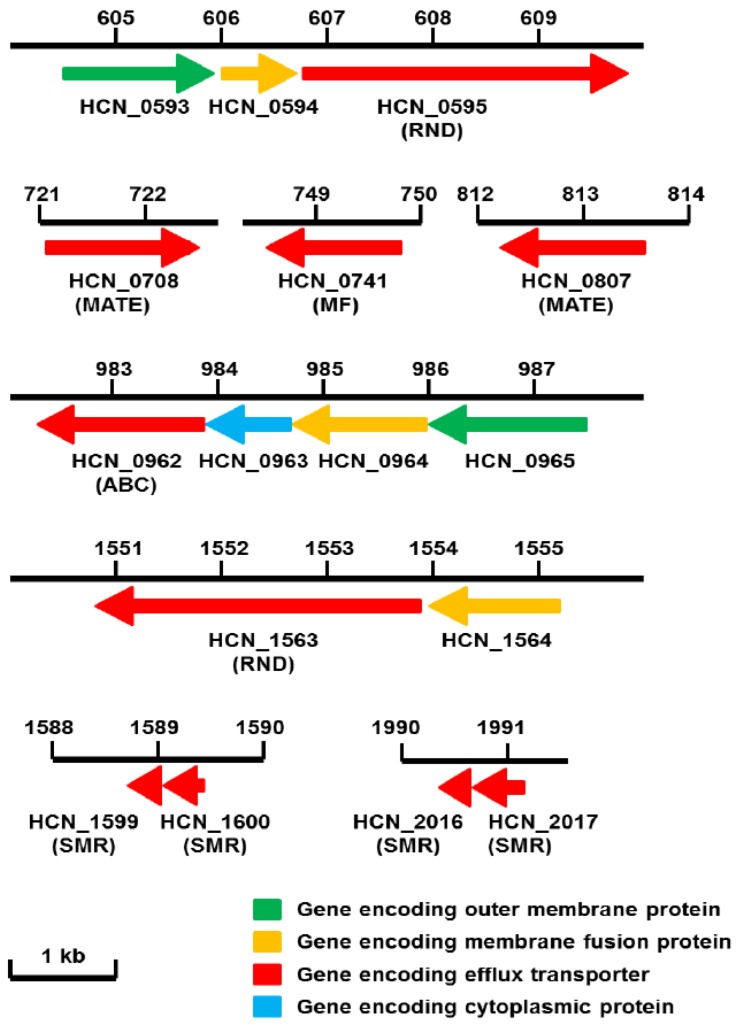
Drug efflux genes encoded in the genome of *H. cinaedi* PAGU 611. Chromosomal positions of drug efflux genes coding for putative inner membrane efflux transporters (red), outer membrane proteins (green), membrane fusion proteins (orange), and cytoplasmic proteins (light blue) are indicated by the kb (kilobase pair) in the *H. cinaedi* PAGU 611 genome [22]. Arrows correspond to the lengths and directions of the genes.

Very recently, we announced the complete genome sequence of *H. cinaedi* PAGU 611 isolated from a case of human bacteremia in Japan [22]. The clinical microbiological aspect of this strain was described as *H. cinaedi*-case 1; strain 923 [3]. Three months after our original report, another group published the sequence of the strain ATCC BAA-847, which was isolated in the 1980s in the USA [23]. The genome sequence of *H. cinaedi* CCUG 18818, although just a whole genome assembly and not complete, is also available from the Human Microbiome Project [24]. *H. cinaedi* PAGU 611 had a threonine to isoleucine mutation at position 84 of GyrA and adenine to guanine at position 2060 in PAGU 611 and ATCC BAA-847 (position 2018 in CCUG 18818) in the 23S rRNA gene, both of which are the same mutations identified in recent ciprofloxacin- and clarithromycin-resistant *H. cinaedi* isolates in Japan [7,8]. In addition to the slow, poor and, sometimes, failed growth described above, genetic tools for *H. cinaedi* are not sufficiently developed to take full advantage of the wealth of information generated by genome sequencing and to elucidate the function of unknown genes identified through sequencing. Fortunately, gene replacement via homologous replacement in *H. cinaedi* is possible by electroporation; however, no complementation system, e.g., a plasmid vector, is currently available for this organism [25]. We identified 10 putative drug transporter genes (2 RND, 1 MF, 2 MATE, 1 ABC, 4 SMR) in the genome of *H. cinaedi* PAGU 611 [22] (Figure 1). All transporters have homologues in *H. hepaticus* ATCC 51449, while only two-fifths are in *H. pylori* 26695 (Table 1). Interestingly *C. jejuni* subsp. *jejuni* NCTC 11168 has, rather, the most homologues (Table 1). Here, we compare the organization and properties of the multidrug efflux systems of *H. cinaedi* with the characterized and uncharacterized pumps available in the database.

## 4. RND Efflux Gene Operons of *H. cinaedi*

We identified two open reading frames (ORFs) belonging to the hydrophobe/amphiphile efflux-1 (HAE1) sub-family [26] of the RND family (locus-tags HCN_0595 and HCN_1563) encoded in the 2.08 Mbp chromosome of *H. cinaedi* PAGU 611 (Figure 1). One consists of three genes (HCN_0593-HCN_0594-HCN_0595) that encode OMP, MFP and RND, respectively, and the other consists of two genes (HCN_1564-HCN_1563) that encode MFP and RND, respectively. The ORFs were obtained from the chromosomes of ATCC BAA-847 and CCUG 18818. Both a three-gene operon (MFP, RND, and OMF) and a two-gene operon (MFP and RND) are genetically common as a multidrug efflux operon, while the latter is functionally associated with an OMF component that is encoded by a separate gene that is physically unattached to the other two members on the chromosome. For example, in *P. aeruginosa* PAO1, *mexAB-oprM* and *mexXY* encode two multidrug efflux pumps (MexAB-OprM and MexXY-OprM, respectively) and contribute to natural antimicrobial resistance [27]. However, three-gene RND-type multidrug efflux operons (e.g., *mexAB-oprM* of *P. aeruginosa* [28] and *cmeABC* of *C. jejuni* [29]) are usually in the order MFP-RND-OMF, unlike *H. cinaedi*, *H. pylori* and *H. hepaticus* [9,30].

## 5. Structure of the RND Components (HCN_0595 and HCN_1563) of *H. cinaedi*

The RND components of RND-type tripartite multidrug efflux pumps determine their substrate specificity [31,32]; therefore, we focused on the structure and function of the RND components of *H. cinaedi* (HCN_0595 (YP_006638872) and HCN_1563 (YP_006235870)), rather than the OMPs or MFPs. BLAST analysis [33] showed that HCN_0595, with a calculated molecular mass of 112 kDa, exhibited strong sequence homology to the uncharacterized RND component of the HH0222 pump (NP_859753, 86% (94%) identity (positive)) of HH0224-HH0223-HH00224 (named HefABC after those of *H. pylori* [9]) of *H. hepaticus* ATCC 51449 and significant homology to the HefC (NP_207402, 58% (78%) identity (positive)) RND component of the HefABC pump of *H. pylori* 26695 [30] (Table 1)and uncharacterized pumps (49%–58% (70%–78%) identity (positive)) of various other *Helicobacter* species (e.g., *H. acinonychis*, *H. cetorum*, *H. mustelae*, *H. bizzozeronii*, *H. bilis*, *H. suis*, *H felis*, *H. pullorum*, *H. winghamensis*, and *H. canadensis*) and other ε-proteobacteria (e.g., *Wolinella succinogenes*). The HefC pump of *H. pylori* was shown to play a critical role in resistance to bile salts and ceragenins, non-peptide mimics of antimicrobial peptides [30]. This pump might also be involved, to some extent, in antimicrobial resistance, including metronidazole [e.g., 34], although genetic evidence for the HefC pump has not been provided [35]. In *C. jejuni* subsp. *jejuni* NCTC 11168, the best-studied organism for efflux systems in ε-proteobacteria, CmeF (YP_002344428), the RND component of the CmeDEF pump [36], but not CmeB (YP_002343803), the RND component of the CmeABC pump, showed significant similarity (38% (59%) identity (positive)) to HCN_0595. The contribution of CmeDEF to intrinsic resistance is likely to be small or secondary compared with that of the major multidrug efflux system CmeABC [36,37]. We could not find any other characterized pumps that were significantly similar to HCN_0595.

**Table 1:** Homologues in the other representative ε-proteobacteria for the putative drug efflux transporters of *H. cinaedi* PAGU 611. Homologues in *H. hepaticus* ATCC 51449, *H. pylori* 26695 and *C. jejuni* NCTC 11168 for the putative efflux transporters of *H. cinaedi* PAGU 611 are shown using BLAST analysis.

Putative drug transporter	Family	Homologue (Identity (%) (Positives (%)))		
		*H. hepaticus*	*H. pylori*	*C. jejuni*
		ATCC 51449	26695	NCTC 11168
HCN_0595	RND	HH0222 (86 (94))	HefC (58 (78))	CmeF (38 (59))
HCN_0708	MATE	HH0167 (81 (90))	HP1184 (48 (73)	Cj0560 (29 (49))
HCN_0741	MF	HH1614 (80 (90))	HP1181 (47 (64))	CmeG (43 (64))
HCN_0807	MATE	HH0031 (76 (87))	HP0759 (40 (62))	
HCN_0962	ABC	HH1856 (87 (93))		Cj0607 (32 (57))
HCN_1563	RND	HH0174 (88 (95))		CmeB (53 (73))
HCN_1599	SMR	HH0508 (59 (74))		Cj1174 (54 (76))
HCN_1600	SMR	HH0509 (61 (73))		Cj1173 (40 (64))
HCN_2016	SMR	HH1452 (61 (73))		Cj0309c (57 (73))
HCN_2017	SMR	HH1451 (56 (69))		Cj0309c (57 (73))

HCN_1563, with a calculated molecular mass of 113 kDa, exhibited strong sequence homology to uncharacterized RND pumps of enterohepatic *Helicobacter* species, including HH0174 (NP_859705, 88% (95%) identity (positive)) of the HH0175-HH0174 pump (named CmeAB after those of *C. jejuni* [9]) of *H. hepaticus* ATCC 51449 and HRAG_01727 (ZP_04580572, 80% (90%) identity (positive)) of *H. bilis* ATCC 43879, but not of gastric *Helicobacter* species, such as *H. pylori* (Table 1). Actually, HCN_1563 exhibited high similarity with major RND multidrug efflux pumps (CmeBs) of *Campylobacter* species (e.g., CmeB of *C. jejuni* subsp. *jejuni* NCTC 11168 (YP_002343803, 53% (73%) identity (positive)) (Table 1). The genome of *H. cinaedi* was the most similar to that of *H. hepaticus* [22], which exhibits a unique combination of features mainly from *H. pylori* and C. *jejuni* [38]. HCN_1563 might be a pump required to survive in the gut environment, but not the gastric environment. The contribution of the CmeABC efflux pump to acquired resistance of *C. jejuni* with target mutations to macrolides and fluoroquinolones has been described [10,39,40,41], which is similar to the ciprofloxacin- and clarithromycin-resistant *H. cinaedi* clinical isolates identified in Japan since 2000 [7,8]. With the exception of *ε-proteobacteria*, the BepE (NP_697326, 55% (73%) identity (positive)) and BepG pumps (NP_699529, 43% (65%) identity (positive)) of *Brucella suis* 1330 are taxonomically classified within *α-proteobacteria* [42], the TtgB pump (YP_006536083, 47% (68%) identity (positive)) of *Pseudomonas putida* DOT-T1E [43] and the AheB pump (YP_857414, 43% (65%) identity (positive)) of *Aeromonas hydrophila* subsp. *hydrophila* ATCC 7966 [44] are noteworthy as very similar pumps. HCN_1563 also exhibited significant similarities, as judged from phylogenetic distance, with well-studied multidrug efflux pumps (AcrB/AcrD/AcrF pumps (41% (61%–64%) identity (positive)) of *E. coli* and MexB/MexF/MexD/MexY pumps (39%–43% (61%–65%)) of *P. aeruginosa* [13,14]. 

**Figure 2:**
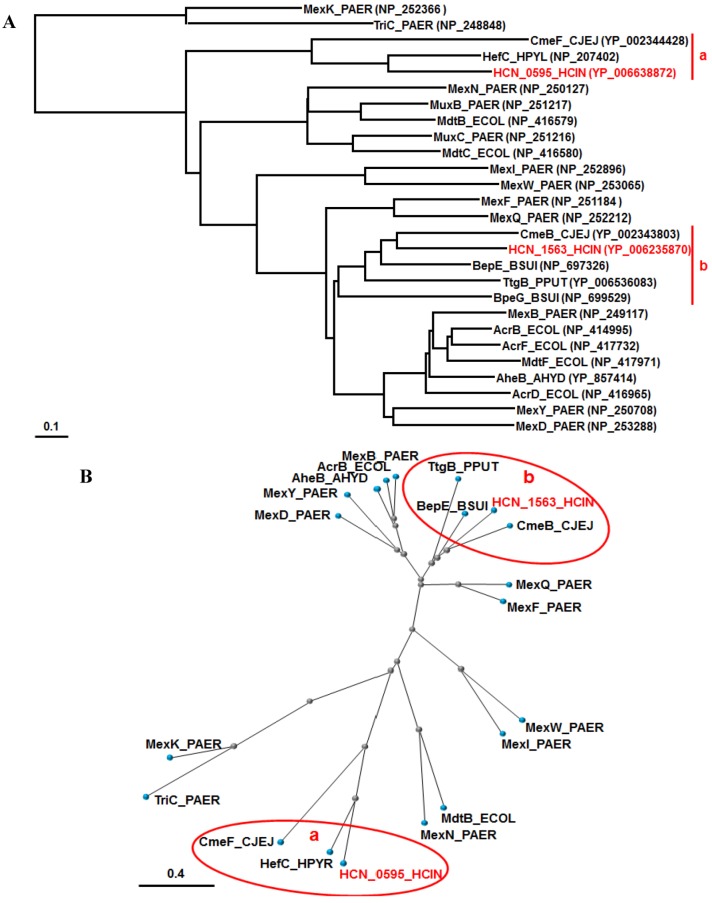
Phylogenetic trees for RND pumps of various bacteria. According to the COBALT program, the trees were constructed using the Fast evolution method and rendered with (**A**) Rectangle and (**B**) Radical. The accession numbers are shown in parentheses. The branches belonging to HCN_0595 and HCN_1563 of *H. cinaedi* PAGU 611 are shown in red and named “a” and “b”, respectively. The proteins are abbreviated (e.g., “AcrB_ECOL” stands for “AcrB of *E. coli*”). Abbreviations; PAER, *Pseudomonas aeruginosa*; CJEJ, *Campylobacter jejuni*; HPYR, *Helicobacter pyroli*; HCIN, *Helicobacter cinaedi*; ECOL, *Escherichia coli*; BSUI, *Brucella suis*; PPUT, *Pseudomonas putida*; AHYD, *Aeromonas hydrophila.*

COBALT analysis [45] of representative RND pumps in Gram-negative bacteria, including all RND pumps from *P. aeruginosa* PAO1 and *E. coli* K12, characterized their relationships, and we focused on two branches containing the two RND pumps of *H. cinaedi* (Figure 2). The branch belonging to the HCN_0595 pump only includes HefC of *H. pylori* and CmeF of *C. jejuni*, while the branch containing the HCN_1563 pump includes not only CmeB of *C. jejuni*, but also the BepE/G pumps of *B. suis* and TtgB of *P. putida* (Figure 2). 

Taken together, we assume that the HCN_0595 pump of *H. cinaedi* plays a similar role to HefC of *H. pylori* and CmeF of *C. jejuni*, while the HCN_1563 pump has a similar role as CmeB of *C. jejuni*. In addition, the two pumps of *H. cinaedi* must play very similar roles to those of *H. hepaticus*, which is not surprising, according to their biological and genomic similarities. Recently, HefA (HH0224), the OMF component of HefABC of *H. hepaticus* ATCC 51449, was shown to be involved in resistance to amoxicillin and some antimicrobials, as well as bile acids [9]. As the authors failed to isolate a mutant RND pump (HH0222 (HCN_0595 orthologue) and HH0174 (HCN_1563 orthologue) in Figure 2), we do not know if the resistance to amoxicillin and bile acids is caused by HH0222 or HH0174, because the HH0174 gene is a two-gene operon, like the HCN_1563 gene [9]. It is noteworthy that the HefC pump of *H. pylori* played a role in cholesterol-dependent resistance in the bile salt-rich enterohepatic environment [30]. Cholesterol enhanced *H. pylori* resistance to various antibiotics, such as clarithromycin, amoxicillin and ciprofloxacin, as well as bile salts (e.g., deoxycholate) [30,46]. It is intriguing to determine if *H. cinaedi* resistance is enhanced by cholesterol and if the RND pumps of *H. cinaedi* play a role in cholesterol-dependent resistance. Actually, *hefABC* of *H. hepaticus* and *H. pylori* and *cmeABC* of *C. jejuni* were inducible by bile acids [9,30,47]. It is of note that CmeABC of *C. jejuni* plays a critical role in colonization *in vivo* [48].

## 6. A Possible Regulator Gene of Multidrug Efflux Systems in *H. cinaedi*

Although cognate regulators (e.g., repressors, activators, or two-component systems) located upstream of the RND efflux genes often exist, no cognate regulator was found upstream or downstream of the RND efflux operons of *H. cinaedi*, *H. hepaticus and H. pylori*. In *C. jejuni*, *cmeR*, which is a transcriptional repressor located immediately upstream of the *cmeABC* operon, encodes a 210 amino-acid protein that shares sequence and structural similarities with the members of the TetR family of transcriptional repressors [49]. BLAST analysis did not identify a homologue of CmeR in the genomes of *Helicobacter* species. Actually, *H. cinaedi* possesses only a small set of genes encoding transcriptional regulators, very similar to *H. hepaticus* [22,38].

Very recently, CosR, an oxidative stress responsive global regulator essential for viability [50], was shown to regulate the *cmeABC* operon negatively by binding directly upstream of *cmeABC* in *C. jejuni* NCTC 11168 [51]. CosR homologues are found mostly in ε-proteobacteria [51]. BLAST analysis showed that a quite similar CosR homologue (HCN_1079, YP_006235418.1) exists in *H. cinaedi* PAGU 611 (74% (86%) identity (positive)) and the strains ATCC BAA-847 and CCUG 18818. This homologue might also be involved in the expression of an efflux gene in *H. cinaedi*. In Gram-negative bacteria, oxidative stress responses are linked to the development of antimicrobial resistance, resulting from the activation of a resistance mechanism in which the RND multidrug efflux system is an important component [52]. For example, exposure to reactive oxygen species, such as peroxide, leads to MexXY-dependent aminoglycoside resistance in *P. aeruginosa* [52,53]. We point out that the putative start codon of all CosR homologues (HCN_1079, HCBAA847_0895, and HCCG_01220) of the *H. cinaedi* strains is TTG, which is a minor start codon [54], and found that an ATG codon located 3 codons before this TTG is also a possible start codon that is preceded by ribosome binding site-like sequences [55].

## 7. *C. jejuni* CmeG Homologue Identified in *H. cinaedi*

CmeG homologues (e.g., HCN_0741 (YP_006235115) of PAGU 611) found in the three *H. cinaedi* strains showed significant homology (43% (64%) identity (positive)) (Figure 1, Table 1). BLAST analysis showed that HH1614 of *H. hepaticus* ATCC 51449 is a strong homologue (80% (90%) identity (positive)) (Table 1). CmeG was shown to function as a multidrug efflux transporter of the MF family that contributes to antimicrobial resistance and oxidative defense (hydrogen peroxide) in *C. jejuni* [56]. Mutations of *cmeG* significantly reduced resistance to various classes of antimicrobials, including ciprofloxacin, tetracycline, gentamicin, ethidium bromide and cholic acid, and overexpression of *cmeG* in the wild-type background increased resistance to fluoroquinolones [56]. CmeG shows significant homology to well-known MF-type multidrug efflux transporters of Gram-positive bacteria, such as NorA of *Staphylococcus aureus* (27% identity) and Bmr of *Bacillus subtilis* (27% identity) [56]. 

## 8. Other Probable Drug Efflux Systems in *H. cinaedi*

Finally, we discuss other probable drug efflux systems found in *H. cinaedi*, although the clinical significance and natural function of their homologues in other characterized bacteria remain unknown. 

**Figure 3:**
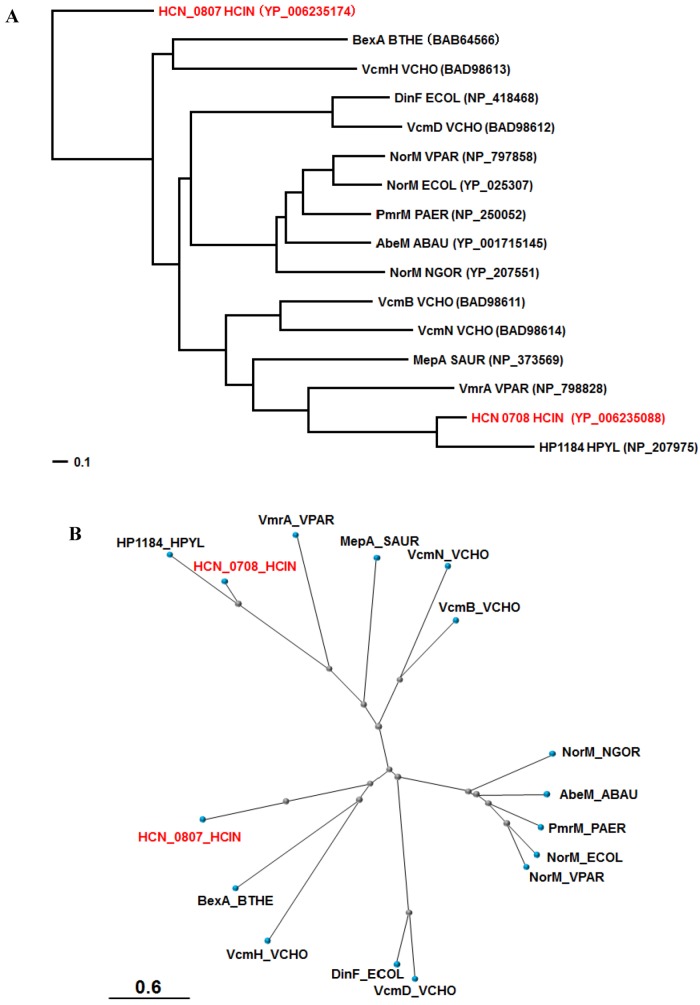
Phylogenetic trees for the MATE pumps of various bacteria. According to the COBALT program, the trees were constructed using the Fast evolution method and rendered with (**A**) Rectangle and (**B**) Radical. The proteins are abbreviated (e.g., “NorM_ECOR” stands for “NorM of *E. coli*”). The accession numbers are shown in parentheses. HCN_0708 and HCN_0807 of *H cinaedi* PAGU 611 are shown in red. Abbreviations; HCIN, *Helicobacter cinaedi*; BTHE, *Bacteroides thetaiotaomicron ; VCHO, Vibrio cholera;* ECOL, *Escherichia coli*; VPAR, *Vibrio parahaemolyticus*; PAER, *Pseudomonas aeruginosa*; ABAU, *Acinetobacter baumannii*; NGOR, *Neisseria gonorrhoeae*; SAUR, *Staphylococcus aureus*; HPYR, *Helicobacter pyroli*.

Two MATE family multidrug efflux family transporters (HCN_0708 and HCN_0807) were found in *H. cinaedi* PAGU 611 and the other two *H. cinaedi* strains (Figure 1). BLAST analysis showed strong homologues of HCN_0708 and HCN_0807 are HH0167 (NP_859698) and HH0031 (NP_859562) of *H. hepaticus* ATCC 51449 (81% (90%) and 76% (87%) identity (positive)), respectively (Table 1). COBALT analysis with MATE pumps characterized in other bacteria showed that HCN_0708 was close to HP1184 of *H. pylori* [57], followed by VmrA of *Vibrio parahaemolyticus* [58], while HCN_0807 seemed unique, but comparably close to BexA of *Bacteroides thetaiotaomicron* [59] and VcmH of *Vibrio cholerae* [60] (Figure 3). Both BexA and VcmH gave resistance to hydrophilic quinolones (e.g., norfloxacin and ciprofloxacin) when expressed in an *E. coli* mutant lacking an *acrB* gene encoding the major RND multidrug efflux pump [59,60].

Two putative SMR family efflux systems (HCN_2017-HCN_2016 and HCN_1600-HCN_1599), both of which encode two SMR components, were found in *H. cinaedi* PAGU 611 and the other two *H. cinaedi* strains (Figure 1). BLAST analysis showed that strong homologues of HCN_2017-HCN_2016 and HCN_1600-HCN_1599 are HRAG_00571-HRAG_00572 (ZP_04582237-ZP_04582238) of *H. bilis* ATCC 43879 (94% (96%) and 98% (99%) identity (positive)) and HH509-HH508 (NP_860040-NP_860039) of *H. hepaticus* ATCC 51449 (59% (74%) and 67% (86%) identity (positive)), respectively (Table 1). Of note, a strong homologue of HCN_2017-HCN_2016 was HH1451-HH1452 (NP_860982-NP_860983) of *H. hepaticus* ATCC 51449 (56% (69%) and 61% (73%) identity (positive)) (Table 1). Both components appear to be necessary for pump activity, e.g., EbrAB and YkkCD of *Bacillus subtilis* [61,62]. BLAST analysis with *E. coli* K12, *P. aeruginosa* PAO1, *B. subtilis* 168 and *Staphylococcus aureus* N315 suggested that HCN_2017-HCN_2016 showed significant similarity to YkkCD (NP_389192 and NP_389193; 43% (60%) and 48% (68%) identity (positive), respectively) of *B. subtilis*, while HCN_1599-HCN_1600 showed significant similarity to MdtJI (NP_416117 and NP_416116; 38% (57%) and 38% (63%) identity (positive), respectively) of *E. coli* K12. YkkCD is a multidrug efflux pump that gives rise to broad specificity, including to cationic (e.g., streptomycin, tetracycline and ethidium bromide), neutral (e.g., chloramphenicol), and anionic compounds (e.g., phosphonomycin), when expressed in *E. coli* [62]. In addition, MdtJI overexpression conferred resistance to deoxycholate when expressed in an *E. coli* mutant lacking *acrB*, a major RND multidrug efflux pump [63], and rescued cell toxicity and growth inhibition due to the over-accumulation of spermidine in a spermidine acetyltransferase-deficient *E. coli* mutant [64].

One ABC family efflux system was found in *H. cinaedi* PAGU 611 (Figure 1). It consists of four genes (HCN_0962-HCN_0963-HCN_0964-HCN_0965) encoding an inner membrane transporter, ATP binding protein, MFP and OMF, respectively, which means that it is an ABC transporter that spans the entirety of the Gram-negative cell envelope. The same efflux system was observed in the two other *H. cinaedi* strains. BLAST analysis showed that HH1856 (NP_861387) of *H. hepaticus* ATCC 51449 was a strong homologue of HCN_0962 (87% (93%) identity (positive)) (Table 1). BLAST analysis with *E. coli* K12 and *P. aeruginosa* PAO1 suggested that HCN_0962 was significantly similar to the inner membrane domains of both MacB (NP_415400; 34% (56%) identity (positive)) of *E. coli* K12 and PvdT (33% (54%)) of *P. aeruginosa* PAO1. MacB and PvdT are inner membrane components of the macrolide-specific ABC transporter MacAB of *E. coli* [19] and of the *de novo* synthesized pyoverdine secretion system PvdRT-OpmQ of *P. aeruginosa*, respectively [65].

## 9. Future Perspective

The genome of *H. cinaedi* possesses probable uncharacterized drug efflux systems consisting of two RND pumps, one MF pump, two MATE pumps, two SMR pumps and one ABC pump, all of which are very similar to those of *H. hepaticus*. Because multidrug efflux pumps have roles in not only bacterial drug resistance, but also in other systems, including virulence and the stress response [52,63], characterizing the multidrug efflux pumps of *H. cinaedi* should lead to the understanding of various physiological aspects of this organism and, ultimately, conquering *H. cinaedi* infections. To do so, it is necessary to develop genetic tools and improve the culture method for this organism, while we can also use multiplex technologies, such as real-time PCR, DNA microarrays, proteomics and metagenomics. In the meantime, each pump can be cloned and characterized in organisms that lack a homologue, such as *E. coli*, *C. jejuni* and *H. pylori*, but some uncertainties will remain. Interestingly, *H. cinaedi* PAGU 611, but not ATCC BAA-847, possesses one plasmid, pHci1 (~23 kbp, 29 predicted coding sequences, of which 27 are hypothetical proteins) [22,23]. As such, it may represent a diamond in the rough that can be developed into a stable shuttle vector, although no replication protein or origin of replication have yet been found in this plasmid. 
